# Skin Cooling to Reduce the Pain Associated with Local Anesthetic Injection; a Randomized Controlled Trial

**DOI:** 10.22037/aaem.v10i1.1562

**Published:** 2022-03-10

**Authors:** Saeed Majidinejad, Farhad Heidari, Amirhosein Famil Chitgarian

**Affiliations:** 1 Department of Emergency Medicine, Isfahan University of Medical Sciences, Isfahan, Iran.

**Keywords:** Lidocaine, Wounds and Injuries, Injections, Anesthesia, Local, Pain, Emergency Service, Hospital

## Abstract

**Introduction:**

Different methods have been proposed for the reduction of the pain caused by the injection of local anesthetics. This study aimed to evaluate the effect of skin cooling on reduction of pain associated with local injection of lidocaine buffered with sodium bicarbonate.

**Methods:**

This randomized controlled trial included 108 adult patients with arm/forearm wounds who referred to the emergency departments. Participants were randomly allocated to two equal groups. Patients in both groups received subcutaneous injection of buffered lidocaine. In the intervention group, an ice cube measuring 2 × 2 × 2 cm (at 0 ° C) in sterile gloves were placed on the wound for 2 minutes before the injection of buffered lidocaine. The primary outcome was severity of pain during lidocaine injection using a visual analog scale (VAS).

**Results:**

One hundred and eight patients were enrolled in the study, 54 in each group. There was no statistically significant difference in age (p = 0.777), sex (p = 0.466), and length of laceration (p = 0.410) between the two groups. The pain scores during lidocaine injection were significantly lower in the intervention group compared to control group (2.39 ± 1.14 vs 4.26 ± 0.94, p < 0.001).

**Conclusions:**

Skin cooling prior to the injection of local anesthetics can significantly reduce the pain caused by local anesthetic infiltration

## 1. Introduction:

Primary repair of wounds is usually performed in the emergency department (ED). Wounds of arms/forearms can occur frequently since the upper extremities are quite often used to ward off serious injuries. The skin of arm/forearm is thin with densely distributed nociceptors resulting in the lower threshold of pain in these areas; therefore, even uncomplicated surgical procedures such as suturing of simple lacerations may require subcutaneous injection of local anesthetics (1, 2). 

Lidocaine, as the most popular anesthetic agent, is used to alleviate procedural pain but paradoxically it can cause pain or discomfort during subcutaneous injection (3-5). For patients undergoing minor surgery such as wound repair, it is often the lidocaine injection that is the most painful part of procedure (2, 3). Administration of local anesthesia for wound repair appears to be the seventh most uncomfortable ED procedure (2). Various methods have been used for relieving the pain caused by local anesthetics injection in previous studies, including warming lidocaine or mixing it with sodium bicarbonate (3). Buffering with sodium bicarbonate or warming will reduce the shelf life of lidocaine from 14 to 7 days requiring additional equipment for storage (2, 3). 

Cooling has been used for analgesia and pain management (6). Ice cubes and cooling sprays have been reported to effectively reduce the pain caused by arterial or venous punctures (7, 8). Pain alleviation for bruises, fractures, bites, sports injuries, sprains, and burns has been achieved with this technique as well (9). Cryotherapy has also been demonstrated to be effective for postoperative pain management when applied preoperatively (10, 11). Pre-cooling for pain reduction in dental procedures is also well described (12). 

Preemptive cryotherapy for relieving the pain caused by subcutaneous injection of lidocaine for the repair of simple lacerations has shown to be effective in wounds occurring in different parts of the body including upper and lower extremities, trunk, and face (2). This study aimed to evaluate the effectiveness of skin cooling on the reduction of pain associated with local injection of lidocaine buffered with sodium bicarbonate.

## 2. Methods:

***2.1. Study design and setting***

This prospective randomized controlled trial was conducted in the emergency department (ED) of Alzahra and Kashani Hospitals in Isfahan, Iran, in 2021. The Ethics Committee of Isfahan University of Medical Sciences (IR.MUI.MED.REC.1398.278) approved the study protocol and it complies with the statements of the Declaration of Helsinki. The trial was registered in the Iranian Registry of Clinical Trials under the number IRCT20180129038549N14. All patients provided written informed consent for participation in the study. 

***2.2. Participants***

All adult patients with acute arm/forearm wounds referred to the ED were enrolled in the study. Inclusion criteria were 18-70 years of age, superficial wound/laceration of arm/forearm (a laceration not involving the fascia or muscle and requiring a one-layer suture), wound surface area ≤5 cm^2^, being alert, and willing to participate in the study. Patients with visual, mental, or verbal disorders, multiple trauma, unstable vital sign, a history of peripheral neuropathy, and a history of an allergic reaction to local anesthetics were excluded. Patients experiencing agitation or irritability caused by cooling during the study were also excluded. 

***2.3. Interventions***

Then patients were divided into two equal groups (intervention group and control group) using the Random Allocation software and simple random sampling method. In the intervention group, an ice cube measuring 2 × 2 × 2 cm (at 0 ° C) in sterile gloves were placed on the wound for 2 minutes and then buffered lidocaine injection was performed. In the control group, no ice cube was used before injecting buffered lidocaine.

Patients in both groups received approximately 10 ml of buffered lidocaine that were fitted with 27‐guage needles. 1% lidocaine hydrochloride mixed with 8.4% Sodium bicarbonate with a ratio of 9 to 1 was used for this study. Since the buffered solution has increased pH, its half-life in room temperature decreases; therefore, the solution was made right before the injection. The buffered lidocaine was injected by an assistant who was blinded to the allocation.

Immediately after injection, each patient was evaluated to rate the pain using the visual analogue scale (VAS) from 0 to 10 by marking a vertical line (0: no pain, 10: the worst possible pain imaginable). In the beginning of the study, patient characteristics (age, and sex) and length of the laceration were recorded. All measurements and data gathering were performed by investigators blinded to randomization.

***2.4. Data analysis***

The Statistical Package for the Social Sciences (SPSS) software (version 25.0, Armonk, NY: IBM Corp.) was used for data analysis. Mean, standard deviation, frequency, and percentages were used to describe the results. The chi-squared test was used to compare sex distribution between groups. Independent t-test was used to compare age and pain scores between groups. P-values ≤0.05 were regarded as statistically significant. 

The sample size was determined as at least 54 participants in each group using the formula for comparison of two means with α=0.05, β=0.2, δ_1_=3.5, δ_2_=7, µ_1_=2, and µ_2_=5 based on the mean of pain scores in two groups form the study by Song et al. (2). 

## 3. Results:

**Figure 1:**
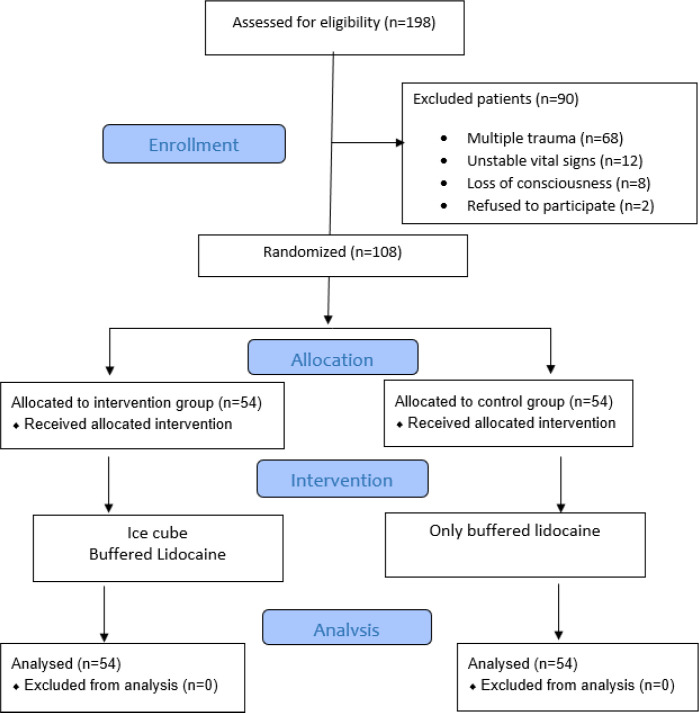
CONSORT flow diagram of the study

A total of 198 patients were evaluated, 108 of whom were included in the analysis; 54 patients in the intervention group, and 54 patients in the control group (Figure 1). The mean age was 43.95±13.14 (19-68 years) years (80.6% male). There was no statistically significant difference regarding age (p = 0.777), sex (p = 0.466) and length of laceration (p = 0.410) between the two groups (Table 1).

The pain scores during lidocaine injection were significantly lower in the intervention group compared to control group (2.39 ± 1.14 vs 4.26 ± 0.94, p < 0.001). No wound complications occurred in either group. Shivering was observed in 2 patients in the intervention group who did not need treatment.

## 4. Discussion:

Based on the findings of the present study; skin cooling prior to the injection of local anesthetics can significantly reduce the pain caused by local anesthetic infiltration. Local anesthetic injection is regarded as one of the most painful components of minor procedures such as superficial wound repair (2-4). The health care providers’ efforts to effectively control pain have been associated with improved patient outcomes, and it is important to find strategies to reduce all pain-related elements of the procedure (13). Pain and anxiety are thought to prevent some patients from receiving any care, including local anesthetic injections (11). There are several factors that can reduce the pain caused by the anesthesia itself and increase patient satisfaction, including selection of the most appropriate solution, proper preparation of the solution, careful selection of the required equipment, preparation of the site of injection, and procedural techniques (14). 

This study demonstrated that local pre-cooling with ice cube can significantly reduce the pain associated with local anesthetic injection. Similarly, Song et al. showed that the median of subjective numeric rating (0-10 scale) was significantly lower in the cryotherapy group compared to the control group (2). Their study was comparable with ours in many aspects including the purpose of local anesthetic injection, the tool used for evaluation of the perceived pain, and the type of local anesthetic. Nevertheless, they assessed the effect of pre-cooling on local anesthetic injection pain at different sites while we only included patients with arm/forearm wounds. Mahshidfar et al., in their study on patients with superficial lacerations, showed that cooling the injection site prior to local anesthetic injection can significantly reduce the pain and discomfort caused by the injection (5). Their results are consistent with the results of the present study.

Soft tissue cooling has also been found to help decrease pain during injection of local anesthetics in children for dental procedures (15). Furthermore, cryotherapy has been shown to be effective for pain reduction in many surgical fields including obstetrics, ophthalmology, orthopedics, otorhinolaryngology, and plastic surgery (9-11). A previous study showed that cryo-analgesia with ice cubes was effective in reducing the pain of local anesthetic infiltration for eyelid surgery (9).

It is not clear exactly how cooling reduces this pain, but it is thought that vasoconstriction and reduced nerve conduction may play an important role (2, 16-19). Generally non-myelinated C fibers and myelinated A-delta fibers are responsible for rapid transmission of pain. Since pain transmission consists of a set of electrochemical reactions and all chemical reactions are slowed down at lower temperatures, nerve conduction velocity can decrease with reducing tissue temperature. In fact, signal conduction through A-delta fibers stops at 10°C (6). Hence, cooling would provide analgesia by decreasing nerve conduction and increasing pain threshold (10, 16). Other underlying mechanisms of cryotherapy that may contribute to pain reduction are reduced inflammation, decreased oxygen demand, limited production of free radicals, and preventing neural plasticity through reducing free nerve ending sensitivity (17, 18).

**Table 1:** Comparing the baseline characteristics of studied cases between groups

**Variables**	**Skin cooling (n=54)**	**Control (n=54)**	**P-value**
**Sex **			
Male	42 (77.8)	45 (83.3)	0.466
Female	12 (22.2)	9 (16.7)	
**Age (years)**			
Mean ± SD	44.31 ± 12.97	43.59 ± 13.41	0.777
**Laceration size (cm)**			
Mean ± SD	3.72 ± 0.25	3.95 ± 0.33	0.410
Pain severity*			
Mean ± SD	2.39 ± 1.14	4.26 ± 0.94	<0.001

Data are presented as mean ± standard deviation or frequency (%). *Based on VAS, visual analogue scale.

## 5. Limitations

A limitation of the current study may be the injection of buffered lidocaine instead of lidocaine alone because adding sodium bicarbonate to lidocaine has been demonstrated to significantly reduce pain from anesthetic injection based on the belief that the acidity of lidocaine is responsible for the pain caused during its local injection (2). Therefore, the application of buffered lidocaine may have decreased the true effect of pre-cooling. Because of the nature of the intervention, blinding of patients was not possible. Evaluation of pain severity via a subjective method (VAS) can be considered as a limitation of the present study. 

## 6. Conclusions:

According to the results of the current study, pre-cooling approximately 2 minutes prior to the injection of the local anesthesia with lidocaine, buffered with sodium bicarbonate, in arm/forearm wounds can reduce the pain caused by injection. However, further studies with larger sample sizes are required to confirm the findings of the current study. 

## 7. List of Abbreviations

- ED: emergency department
- VAS: visual analogue scale

## 8. Declarations:

### 8.1. Availability of data and materials

The datasets used and/or analyzed during the current study are available from the corresponding author on reasonable request. 

### 8.2. Competing interests

The authors declare that they have no competing interests.

### 8.3 Funding

This study was financially supported by Isfahan University of Medical Sciences. 

### 8.4 Author's contributions

S.M., F.H., and A.F.C.; Contributed to conception, study design, and data collection and evaluation. F.H. and A.F.C.; Contributed to statistical analysis, and interpretation of data. F.H. and A.F.C.; Drafted the manuscript, which was revised by S.M.. All authors read and approved the final manuscript.

### 8.5. Acknowledgments

We would like to express our sincere gratitude towards the personnel of the emergency departments of Alzahra and Kashani Hospitals, Isfahan, Iran. 
